# Mesenchymal Stem Cell Therapy for Cardiac Inflammation: Immunomodulatory Properties and the Influence of Toll-Like Receptors

**DOI:** 10.1155/2013/181020

**Published:** 2013-12-10

**Authors:** F. van den Akker, S. C. A. de Jager, J. P. G. Sluijter

**Affiliations:** ^1^Department of Cardiology, University Medical Center Utrecht, Heidelberglaan 100, 3584 CX Utrecht, The Netherlands; ^2^ICIN—Netherlands Heart Institute, Catharijnesingel 52, 3511 GC Utrecht, The Netherlands

## Abstract

*Background*. After myocardial infarction (MI), the inflammatory response is indispensable for initiating reparatory processes. However, the intensity and duration of the inflammation cause additional damage to the already injured myocardium. Treatment with mesenchymal stem cells (MSC) upon MI positively affects cardiac function. This happens likely via a paracrine mechanism. As MSC are potent modulators of the immune system, this could influence this postinfarct immune response. Since MSC express toll-like receptors (TLR), danger signal (DAMP) produced after MI could influence their immunomodulatory properties. *Scope of Review*. Not much is known about the direct immunomodulatory efficiency of MSC when injected in a strong inflammatory environment. This review focuses first on the interactions between MSC and the immune system. Subsequently, an overview is provided of the effects of DAMP-associated TLR activation on MSC and their immunomodulative properties after myocardial infarction. *Major Conclusions*. MSC can strongly influence most cell types of the immune system. TLR signaling can increase and decrease this immunomodulatory potential, depending on the available ligands. Although reports are inconsistent, TLR3 activation may boost immunomodulation by MSC, while TLR4 activation suppresses it. *General Significance*. Elucidating the effects of TLR activation on MSC could identify new preconditioning strategies which might improve their immunomodulative properties.

## 1. Introduction

Ischemic heart disease occurs in approximately 40% of the population above 40 years and is the leading cause of death worldwide [1]. During ischemia a shortage of oxygen and nutrients is present in the heart, leading to apoptosis and necrosis of cardiomyocytes and endothelial cells [2–4]. Subsequent restoration of blood flow is currently the most effective therapy, yet results in additional damage of the myocardium, which is also known as ischemia/reperfusion injury (I/R injury) [5–7]. Both ischemia and reperfusion-induced cell death trigger a strong inflammatory reaction [8–10]. After initiation, this response is propagated by the damaged cells and matrix via the release of chemokines, cytokines, and a variety of endogenous proteins, referred to as danger-associated molecular patterns (DAMPs). DAMPs can subsequently bind toll-like receptors (TLR) on immune and other cells to activate them, resulting in a strong inflammatory environment. This results in an influx of neutrophils, subsequently followed by infiltrating monocytes and lymphocytes [10]. The immune response is essential to clear up the debris caused by the infarct, but also to initiate the wound healing process and the formation of proper scar tissue [10, 11]. The balance between inflammatory and reparative phases is delicate and needs proper fine-tuning in order to prevent excessive inflammation or inadequate stimulation of repair. Eventually this leads to adverse remodeling and subsequently decreased heart functions [5, 12, 13].

The resolution of postinfarct inflammation is considered to be an active process, influenced by factors released by the matrix and local cells, including surviving cardiac cells and infiltrated immune cells [13]. As the regulation of the inflammatory reaction seems inefficient after massive damage, a special interest has developed for the induction of anti-inflammatory or regulatory subtypes of immune cells. This includes alternatively activated (type 2) macrophages and regulatory T-cells, both of which have been claimed to control the progression of the immune response. In time, sturdy scar tissue forms in the damaged areas. The matrix deposition in the scar impedes optimal contraction of the heart, resulting in further loss of cardiac function, which can progressively develop into heart failure [9, 14–17]. Activation of autoreactive T-lymphocytes has been reported at later stages. This negatively influenced cardiac remodeling and cardiac function long after the initial infarction response had occurred [18–21]. This indicates that the influence of immune cells persists long after the initial phase of damage. No curative therapy for heart failure exists besides cardiac transplantation or assistant devices, which is not without risk and many patients die waiting for a heart due to a lack of donor organs [1].

Although the effect of inflammation after MI has been known for many decades, no treatment options currently exist in a clinical setting to properly control this response [10]. Different avenues of treatment have been thoroughly investigated. General suppression of the whole immune system, using cortisone or cytostatic drugs, has shown detrimental effects on overall survival in both animal models and clinical studies. These treatments inhibited scar formation, which greatly increased the chance of cardiac rupture [22–26]. Similar results were found in studies investigating the use of nonsteroidal anti-inflammatory drugs (NSAIDs), where decreased wound healing led to fragile scar formation and decreased survival [27]. Depletion studies for circulating immune cells demonstrated that macrophages were essential for initiating healing after myocardial infarction [12, 28]. Meanwhile, depletion of neutrophils showed a reduction in final scar size without adverse effects on cell survival or cardiac function, demonstrating that their role was unnecessary for healing and only led to additional damage [12, 29–31]. Additionally, a recent rat study investigated T-cell depletion after MI, using antithymocyte globulin to induce T-cell apoptosis. They showed smaller infarcts with reduced remodeling, maintenance of cardiac function, and increased neoangiogenesis [32]. Depletion of B-cells using an anti-CD20 antibody also has beneficial effects on infarct size and heart function, by limiting myocardial inflammation [33]. These studies are only the first steps towards understanding, as for other cells of the immune system or their interactions the role in clearing of cardiac cell debris and stimulation of reparative responses remains largely unknown. What we have learned from these experiments, however, is that a general suppression of the immune response is not a therapeutic answer for modulating post-MI processes.

Recently, mesenchymal stem cells (MSC) emerged as potent modulators of the immune system. Interactions with several cells of the immune system have been described and many reports showed that MSC suppress white blood cells or trigger them into specific anti-inflammatory subsets. Treatment of post-MI inflammation using MSC could therefore provide a new approach of modulating the immune response, shifting the balance towards the reparative phase and reducing inflammation. Although these hypotheses seem to hold for the ideal *in vitro* situation, during post-MI inflammation many danger signals are released which will trigger toll-like receptors (TLR) not just on immune cells, but also on the injected MSC. The effect of TLR activation on MSC function is still largely unknown. It is possible this influences the MSC paracrine signaling capacities, thereby altering their ability to suppress the immune system. This review will focus first on the effects of MSC on the different types of immune cells after MI. Subsequently an overview will be provided of the current knowledge on the effects of DAMP-mediated TLR activation on MSC functioning.

## 2. Stem Cell Therapy against Cardiac Inflammation

Cardiac cell transplantation therapy is a new treatment option using stem cells for myocardial repair [34, 35]. The goal was to stabilize or reverse progressive heart failure by replacing myocardial scar tissue with contractile cells. Stem cells transplanted in the heart are suggested to reduce initial damage after infarction, promote activation of the endogenous regenerative potential of the heart, and integrate in the regenerated tissue [35, 36]. However, despite favorable results on cardiac function obtained in both animal and human studies, only few stem cells were reported to survive in the heart upon injection [34, 37–41]. This indicates that stem cell differentiation and direct contractile contribution are at most a minor explanation for the observed myocardial effects. The release of supportive or paracrine factors by the injected cells is more likely to be responsible—a theory called the paracrine hypothesis [42, 43].

Mesenchymal stem cells (also known as mesenchymal stromal cells or mesenchymal progenitor cells) are a heterogeneous group of stromal cells, which can be isolated from nearly all tissues of mesodermal origin. They are most prevalent in the bone marrow and adipose tissue, but can also be isolated from umbilical cord blood, placenta, dental pulp, and synovium [36, 44, 45]. Despite ongoing efforts, no single marker has yet been found that characterizes a pure MSC population with a homogenous functional profile. MSC are therefore characterized and defined by the minimal criteria described by the International Society for Cellular Therapy [46]. These criteria include (1) adherence to plastic, (2) expression of surface markers CD105, CD73, and CD90, while lacking the expression of CD45, CD34, CD14, or CD11b, CD79alpha or CD19, and HLA-DR surface molecules, and (3) differentiation *in vitro* into osteoblasts, adipocytes, and chondroblasts. In addition to these criteria, differentiation into hepatocytes and cardiomyocytes has been described. However, the *in vivo* occurrence of cardiomyocyte differentiation is rare and is *in vitro* only effective in young cell sources [36, 42, 47, 48].

MSC are especially known for their secretion of paracrine factors, which have beneficial effects on angiogenesis, cell survival, and inflammation. MSC have been shown to regulate the activation and differentiation of many cells of the immune system, including T-cells, B-cells, NK cells, monocytes, dendritic cells, and neutrophils [10]. MSC transplantation is considered safe and has been widely tested as treatment for neurological, immunological, and cardiovascular diseases with promising results [45]. Animal and clinical studies using MSC therapy after MI reported beneficial effects, such as increased ejection fraction and reduced remodeling. However, cell retention in the heart is declining rapidly, with only 10% present after four hours and approximately 1% 24 hours after injection [36, 49, 50]. No long term engraftment and subsequent vascular differentiation have been reported [36]. Interestingly, currently there are about 40 registered trials investigating the effect of MSC therapy for cardiac disease only (clinicaltrials.gov) and many more exist for other diseases, based on their paracrine effectiveness.

## 3. Modulation of the Immune System by MSC

**Figure 1:**
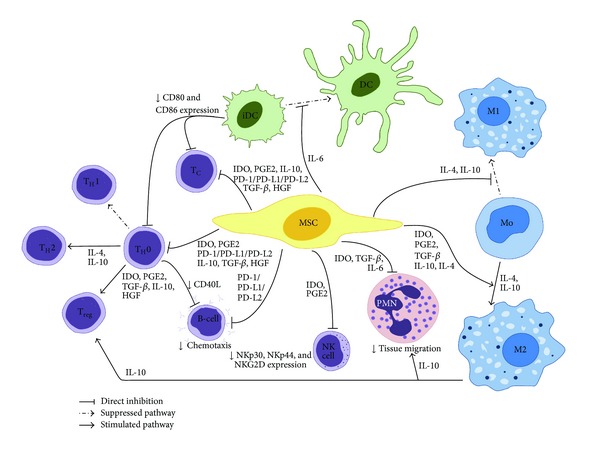
Schematic overview of the interactions between MSC and the immune system. Mesenchymal stem cells influence the functioning of many immune cells. Via multiple possible pathways MSC suppress proliferation of both helper (T_H_) and cytotoxic T-cells (T_C_). In addition, differentiation to T_H_2 and regulatory T-cells (T_reg_) is triggered, resulting in an anti-inflammatory environment. Maturation of immature dendritic cells (DC) is inhibited via IL-6, blocking upregulation of CD40, CD80, and CD86, which in turn can reduce T-cell activation. Monocytes are triggered by MSC to differentiate towards the M2 phenotype. Different mechanisms appear to be involved in this process, amongst which IDO, TGF-*β*, IL-10, and PGE2 are the most important ones. IL-10 produced by these M2 macrophages can boost the formation of  T_reg_, while reducing tissue migration of neutrophils. Neutrophils (polymorphonuclear granulocytes; PMN) are allowed a longer life span by MSC-derived IL-6, while ROS production is decreased. Natural killer cell (NK cells) proliferation is suppressed, as well as cytotoxic activity and cytokine secretion. B-cell proliferation is inhibited and the production of antibodies is reduced.

The discovery that MSC could modulate the immune system was initiated over a decade ago when it was observed that MSC abrogated T-cell proliferation *in vitro* [51]. These observations were quickly transferred to the clinic, where treatment of patients with therapy-resistant acute severe graft-versus-host-disease (GVHD) improved after multiple MSC infusions [52, 53]. In the next phase, MSC were administered simultaneously with hematopoietic stem cells (HSC) to reduce the chances of developing GVHD [54]. The successes obtained in these studies sparked investigations into MSC therapy against graft rejection and autoimmune disease, as both conditions also depend heavily on T-cell activation [55–57]. In the vast majority of these studies, MSC therapy had a favorable effect on inflammation status, disease progression, and functional outcome of the different organs [58–63]. Most research on the immunomodulatory properties of MSC have focused on their interaction with T-cells. To better understand the interactions between MSC and different immune cells, a short overview of the current knowledge will be given for each cell type and is also summarized in Figure 1.

### 3.1. T-Cell Proliferation

T-cells are a heterogeneous group of cells, consisting of many subtypes of which the T-helper cells (T_H_-cells; CD4+), cytotoxic T-cells (T_C_-cells; CD8+), and the regulatory T-cells (T_reg_; CD4+ or CD8+, CD25+ FoxP3+) are best known. Both T_H_  and T_C_-cells recognize a specific antigen, but while T_C_-cells directly induce apoptosis of the cell displaying that particular antigen, T_H_  cells mobilize macrophages and B-cells to attack the antigen-displaying cell. T_reg_  are regulators of the immune response and capable of terminating T-cell mediated immunity. Upon MI, antigen-specific T-cells form against endogenous cardiac myosin and actinin, which leads to a continuous assault of T_H_-cells and T_C_  cells on the remaining myocardium [18, 19, 64].

Several authors showed that MSC are quite potent suppressors of T-cell proliferation, although there is a lot of donor variability [51, 65–67]. As shown in Figure 1, MSC affect both T_H_- and T_C_-cells, by inducing cell cycle arrest of the T-cells in the G0/G1 phase [68]. Many different pathways were found to play a role in this interaction between MSC and T-cells, of which most studied are indoleamine-pyrrole 2,3-dioxygenase (IDO) and prostaglandin E2 (PGE2) [10]. IDO is an enzyme involved in the tryptophan metabolism, which is upregulated in MSC in coculture with T-cells. This leads to tryptophan depletion and local accumulation of metabolites KYNA and kynurenine, all of which are thought to be able to reduce T-cell proliferation [69]. Alternatively, induction of COX-2 expression also occurs in these cocultures resulting in increased secretion of PGE2, thereby inhibiting T-cell proliferation [67, 70]. Another pathway possibly involved is the interaction of inhibitory molecule programmed death 1 (PD-1) and the ligands PD-L1 and PD-L2 [71]. The PD-1/PD-L1/PD-L2 pathway is a regulatory mechanism which controls T-cell-receptor-mediated lymphocyte proliferation and cytokine secretion [71]. MSC expressing PD-L1 or PD-L2 can activate the PD-1 receptor on the target T-cell. This results in a decrease in production of proinflammatory cytokines IFN-*γ*, TNF-*α*, and IL-2 and subsequent T-cell cycle arrest [65, 71]. Another way in which T-cells could be kept inactive is related to the (inducible) expression of MHC (or HLA) molecules on MSC. With these molecules, MSC can play the role of antigen-presenting cell, which would normally activate T-cells [72]. However, due to the absence of an indispensable costimulatory signal from CD80, CD86, or CD40, T-cells might go into anergy instead of being fully activated [45, 73, 74]. In this state, T-cells are still alive, yet unable to be activated and therefore unable to mount a functional immune response.

### 3.2. T-Cell Differentiation

MSC are also able to influence differentiation of T-cells into different subtypes. In addition to the aforementioned pathways, several paracrine factors including HGF, TGF-*β*1, IL-6, and IL-10 have been implicated in this process, although the exact mechanisms still remain unknown [10]. MSC suppress the formation of T_H_1 and T_H_17 lymphocytes, which are essential for the activation of cytotoxic T-cells and the boost of phagocytic capacity of neutrophils and macrophages [75, 76]. Meanwhile, MSC enhance the formation of T_H_2 lymphocytes, which have a more immunotolerant phenotype and produce large amounts of IL-4 and IL-10 [10, 76, 77]. Although these T_H_2 cells normally induce B-cells, there are reports that the costimulatory molecules are downregulated on the T_H_  cells, resulting in a reduction in B-cell activation [78]. Besides reducing T-cell proliferation, MSC also induce formation of regulatory T-cells [75, 76]. This provides a negative feedback loop for T-cell activation and proliferation and helps to regain a tolerance for autoantigens, such as myosin [79]. These regulatory T-cells are suggested to be formed via IDO-expression, secretion of PGE2, and TGF-*β* by interacting MSC. Interestingly, an increase in regulatory T-cells has been shown to attenuate ventricular remodeling after MI [80].

### 3.3. NK Cells

Natural Killer (NK) cells are the innate immune system's subtype of cytotoxic lymphocytes. They usually react in response to viral antigens presented on MHC-I molecules, but can also recognize and lyse stressed cells, which many cardiac cells are shortly after MI [81]. MSC can suppress the proliferation of NK cells, as well as reduce the cytotoxic activity and pro-inflammatory cytokine profile (Figure 1) [82]. Proliferation of NK cells is sharply reduced in the presence of both IDO and PGE2, thereby pointing to the possible synergistic effect of these two pathways [82–84]. MSC can also downregulate the NK activating receptors NKp30, NKp44, and NKG2D [83]. As NK receptors are correlated with the function of the NK cell, the downregulation of activating receptors leads to an altered cytotoxic activity and reduces secretion of pro-inflammatory cytokines [83]. The reduction in IL-2 and IFN-*γ* secretion leads to further suppression of NK cell proliferation [68].

### 3.4. B-Cells

B-cells are part of the adaptive immune response and responsible for the production of antibodies during inflammation. The antibodies cover the cell displaying the specific antigen and allow easy engulfment by phagocytic cells, such as macrophages and neutrophils [85]. After MI, mature B-cells release Ccl7 to attract the pro-inflammatory M1 macrophages to the heart, which decreases cardiac function by enhancing tissue injury [33]. MSC were found to arrest B-cells in the G0/G1 phase, while simultaneously reducing the chemotactic capacity of these cells, as depicted in Figure 1 [68, 74, 86, 87]. How this is exactly regulated remains unclear. MSC can interact with B-cells via the PD-1 pathway as seen for T-cells, hereby reducing activation and proliferation of B-cells [71]. The costimulatory molecule CD40L is mainly present on activated T-cells and plays a role in B-cell activation [77]. If this costimulatory signal is not obtained, B-cells activation will be reduced and antibody secretion will diminish. A reduction in T_H_-cell activation by MSC, and especially the existence of T-cell anergy, could lead to decreased B-cell activity *in vivo* [45, 88]. Finally, some research showed that MSC were able to suppress the production of antibodies by B-cells [86]. It is important to note here that some reports have also been published that MSC stimulate B-cell proliferation and differentiation [89, 90].

### 3.5. Dendritic Cells

Dendritic cells (DC) are the most potent antigen presenting cells of our immune system and after MI they present cardiac antigens, which activate the adaptive immune system [91]. Coculture of MSC with DC progenitors, whether CD34+- or monocyte-derived, prevented differentiation into mature DC, despite the fact that cells were grown in lineage-specifying growth conditions [84, 92–94]. MSC also blocked maturation of DC, leading to a reduced expression or absence of antigens and co-stimulatory molecules CD40, CD80, and CD86, subsequently necessary to activate T-cells (Figure 1) [84, 93, 95]. This process is at least in part regulated via secretion of IL-6 by MSC [60]. MSC induce the production of IL-10 while suppressing IL-2, IL-12, IFN-*γ*, and TNF-*α* by DC, resulting in impaired maturation, migration, antigen capture, and processing [65, 68, 92]. These cytokines are also crucial for the activation of lymphocytes, which was therefore impaired as well. This suggests that MSC may induce a suppressive phenotype of DC which reduced the effector T-cells, but enhanced regulatory T-cell responses [45, 68, 87, 92, 96].

### 3.6. Monocytes/Macrophages

Monocytes, which can differentiate into tissue macrophages, have a dual role in inflammation, and tissue repair. After MI, two major subsets of macrophages can be found in the heart at different time points. Shortly after MI, the classically activated M1 macrophage (inducible nitric oxide synthase (Nos2, iNOS), MHC Class II, CD80, CD86) is present in the heart, which is strongly associated with the clearing of debris, inflammation, and the production of pro-inflammatory cytokines, such as IL-1*β*, TNF-*α*, and IFN-*γ* [97]. After about five days the more prevalent type has switched to the alternatively activated M2 macrophage (Arginase 1 (Arg1); macrophage mannose receptor (Mrc1, CD206); Macrophage scavenger receptor (Msr1, SR-A, CD204)) [97]. This macrophage subtype has an anti-inflammatory phenotype, reducing the release of pro-inflammatory cytokines, while stimulating cardiac reparative pathways, scar formation, and angiogenesis [10, 97–99]. In the presence of MSC, differentiation of macrophages into the M2 subtype was boosted (Figure 1). Many pathways have been indicated in this process, such as IDO, PGE2, and MSC-derived IL-4 and IL-10 [67, 100–102]. MSC also secrete TGF-*β*1, which together with PGE2 were found to reduce the production of pro-inflammatory cytokines by the macrophages, such as IL-1*β*, IL-6, TNF-*α*, and IFN-*γ* [101, 102]. Meanwhile, anti-inflammatory cytokine IL-10 was strongly increased, which in turn is said to boost formation of regulatory T-cells [100, 101]. No negative effects on macrophage phagocytosis were observed in the presence of MSC, meaning their debris-clearing functions were still intact [13, 102].

### 3.7. Neutrophils

Neutrophils kill microorganisms and infected cells by production of reactive oxygen species (ROS) and clearance of the subsequent debris. They are also activated in response to local chemokines and DAMPs after sterile tissue damage, such as MI [68]. Within an hour an influx of neutrophils in the heart is visible and they remain the most prominent cell type for 1-2 days [10]. MSC produce high levels of IL-6, which activates STAT3 transcription factors, resulting in a longer life span of the neutrophils, as indicated in Figure 1 [10]. Although this appears counterintuitive at first, IL-6 also attenuates the neutrophil respiratory burst, so the neutrophils are less harmful to their environment [103]. MSC are able to suppress the degranulation of the enzyme—containing granules of neutrophils. Among others, IDO is found to inhibit the secretion of defensin-*α* (also known as human neutrophil peptide 1–3), which is stored in secreted granules of the neutrophils and has various pro-inflammatory characteristics which can become cytolytic [104] at high concentrations. The effect of MSC on neutrophil tissue migration remains unclear, with few contradicting reports [103, 105, 106]. PGE2 produced by MSC stimulates monocytes and macrophages to produce IL-10, which can prevent neutrophils entering damaged tissue [77, 100]. Likewise, TGF-*β* and IL-10 trigger endothelial cells to reduce their E-selectin expression, which is essential for immune cell extravasation [13, 107].

Even though in most literature researchers try to identify one major pathway which regulates immunomodulation, it is more likely, considering the heterogeneity of the MSC and the numerous parallel systems in immunology, that a combination of pathways provide the optimal effect [10].

The effects described above make MSC appear to be an ideal anti-inflammatory effector, but most of these studies have been performed *in vitro* under artificial inflammatory conditions. When considering using these cells against cardiac inflammation, it is necessary to investigate the environment these cells will encounter after injection. Especially when injected or infused in the heart shortly after MI, MSC will be surrounded by an unfavorable pro-inflammatory environment [5, 10]. What the effect is of this inflammatory environment on the functions of MSC and their effects is worth looking into before commencing large scale clinical trials.

## 4. Effects of TLR-Signaling on MSC

The innate immune system is constantly surveying the body for the presence of so-called “pathogen-associated molecular patterns” (PAMPs), which are detected by highly conserved receptors known as Pattern Recognition Receptors (PRR). Binding of a PAMP to one of these receptors triggers the activation of signaling pathways, ultimately leading to the activation of transcription factors, mainly NF-*κ*B. Subsequently, this leads to inflammatory cell maturation and activation and the production of inflammatory cytokines and chemokines [44, 108, 109]. Toll-like receptors (TLR) are among the best-described receptors of these PRR. TLR are type I transmembrane glycoproteins expressed by many cell types [44, 110, 111]. In addition, intracellular TLR exist which recognize nucleic acids, such as RNA or DNA of pathogens. Receptor activation can control cell surface expression levels, allowing for both positive and negative regulation [112]. In humans, ten different analogues of TLR exist (TLR1-10), while mice express TLR 1–13 [105]. Each receptor is activated by its own specific set of ligands, resulting in the recognition of a wide variety of ligands [108, 113]. In the cell, the TLR-domain interacts with several adaptor molecules (MyD88, TIRAP, TRIF, and TRAM). Activation of TLR leads to nuclear localization of NF-*κ*B, resulting in the transcription of various chemokines, cytokines, and several genes involved in cell maturation [108, 109]. The resulting immune response is intended to clear the pathogen and activate the repair mechanisms of the injured tissue. Interestingly, TLR do not only get activated in response to pathogen challenges, but also in response to signals released during sterile tissue damage, for example, due to ischemia upon MI [36, 114, 115]. These signals are called damage-associated molecular patterns (DAMPs) and include heat shock protein (HSP) 60 and 70, fibronectin extra domain A (-EDA), uric acid, oxidized LDL, intracellular components of fragmented cells, hyaluronan fragments, members of the S100 protein family, eosinophil-derived neurotoxin, myeloid-related proteins-8 and 14, and human defensin-3 [15, 44, 116].

TLR are not only present on immune cells but on a variety of other cell types, including all cardiac cell types, epithelium, and mesenchymal stem cells [108, 116, 117]. MSC express several TLR and it is essential to determine whether MSC capacities might be altered after stimulation of TLR in response to DAMPs released upon MI [118]. Although there are minor disagreements, it is generally accepted that TLR 1–6 are present on human MSC from different origins, such as the bone marrow, adipose tissue, umbilical cord blood, dental pulp or follicle, and Wharton jerry's MSC [44]. Meanwhile, reports on TLR7-10 are less consistent [44, 108, 109, 119–123]. The presence of TLR7, TLR9, and TLR10 on human bone marrow derived MSC has been reported by some groups, while expression of TLR8 has never been detected [108, 122, 123]. Murine MSC were found to express all TLR mRNA, except for TLR9 [123]. There appear to be only small differences in TLR expression between humans, and mice MSCs. Although this would hint that results might be extrapolated from murine to human studies, one should keep in mind that complex immunological processes might still mechanistically run differently.

Variations in expression between MSC from the same origin can of course be due to donor variations, cell isolation method, culture conditions, and whether RNA or protein expression was measured. For example, Delarosa and Lombardo found that hypoxia caused an increase in expression of TLR 1,2,5,9, and 10 in MSC [108], while Tomchuck et al. did not find any effect of hypoxia on TLR levels [120]. In an inflammatory environment TLR2, 3, and 4 appear to be upregulated on MSC, while TLR6 expression decreases slightly [119]. Of all TLR expressed by MSC, TLR3 and TLR4 have the highest expression, making them an interesting subject for study [109, 121, 123, 124]. Upon TLR-stimulation on MSC, different processes have been studied that could be affected for their functional effects, including migration, proliferation, and differentiation, but also their immunosuppressive potential.

### 4.1. Proliferation, Differentiation, and Migration

Most studies investigating the effect of TLR stimulation on MSC found little to no effect on proliferation. In two studies, a slight reduction in MSC proliferation was found after TLR9 activation by using CpG-ODN and TLR3 activation by poly(I:C) [113, 125]. Another *in vitro* study demonstrated increased proliferation upon TLR2 stimulation with Pam3Cys, while blocking all differentiation [122]. Unfortunately, these three studies used MSC from different origins, namely, umbilical cord [113], adipose tissue [125], and bone marrow [122], which made direct comparisons harder. *In vivo* murine TLR4 knockout (KO) MSC were found to have a higher proliferation rate than their wild-type (WT) counterparts [118], while MSC propagation from TLR2 KO mice was reduced [126].

In addition to proliferation, differentiation is one of the most important hallmarks of MSC. In these studies contradicting results were obtained with regard to differentiation, ranging from no effect to general suppression of differentiation. [109, 122, 124]. Osteogenic differentiation could be increased by activation through LPS, PGN, Pam3CSK4, and poly(I:C) [125, 127–129]. Chondrogenic differentiation in response to TLR signaling could be decreased via poly(I:C), or increased via Pam3CSK4, or remain unchanged via poly(I:C) and LPS [109, 124, 128]. Lastly, adipogenesis remained undisturbed in many studies, although a few studies showed suppression of differentiation after stimulation of TLR2, TLR3, and TLR4 [109, 113, 122, 124, 125, 127, 128]. Interestingly, TLR3 activation has also once been described to induce adipogenic differentiation [113].

Relatively few studies investigated the effect of TLR stimulation on MSC migration. MSC are known to home damaged tissues, which permits parenteral administration, so changes in migratory capacity could be very important. Studies show that TLR activation with poly(I:C), LPS and CpG-ODN improved MSC migration [120, 124, 130], although this effect was temporal for TLR3 and TLR4 and this was no longer noticeable at 24 h [124].

### 4.2. Immunomodulation *In Vitro*

Similar to the other MSC cell processes, contradicting effects of TLR stimulation on immune-modulatory capacities of MSC were reported. Since TLR3 and TLR4 are highly expressed on MSC, most studies focused on them but currently the role of these two molecules in immunomodulation by MSC is still largely unknown. In the few completed studies only T-cell proliferation was investigated as a measure of immunomodulation by MSC, despite the known interactions with nearly all cells of the immune system. TLR4 activation of MSC by LPS reduced immunomodulatory abilities of MSC in a small majority of the studies [109, 119, 124, 131], although other studies found no effect of TLR4 activation [113, 127, 132] and other studies found an improvement in immunosuppression [121, 131]. Of special interest is the study by Tomic et al. [131]. They found that immunosuppression by MSC derived from dental follicle was boosted following LPS exposure, while it was inhibited in dental-pulp-derived MSC. Although both cells fulfilled all the MSC characteristic requirements, the origin of the cell strongly influenced the effect of LPS on the paracrine potential.

Activation of TLR3 by poly(I:C) resulted in the majority of studies in an increase of immunosuppressive capacities of MSC [113, 120, 121, 124, 131], although some studies also reported no effect [127] or a decrease in suppressive capacity [109, 119]. While some claimed that immunosuppressive pathways, including IDO and PGE2, were induced in BM-MSC [121], other reported these to be reduced [119]. Studies with other ligands indicated that activation of TLR9 can augment immunosuppressive capacities [113], while TLR2 activation appeared to have no effect [122].

In conclusion, there are many inconsistent results regarding the role of TLR on MSC on their immunomodulatory potential. Based on the reports published so far, activation of TLR3 by poly(I:C) might have beneficial effects, while TLR4 activation could slightly decrease immunomodulative functions. It is important to note, however, that the T-cell suppressive capacity of MSC varied strongly between studies (between ±20 and ±80% in untreated conditions) and the effects of TLR activation were often minor, with only few exceptions [124].

### 4.3. Immunomodulation *In Vivo*

Naturally the normal physiological environment and the cross-talk between different cell types are absent in *in vitro* studies. To get a better idea of the effect of TLR signaling in MSC after MI, a small number of animal models were examined. Acute ischemia reperfusion injury was induced in a TLR-2 KO rat in an *ex vivo* isolated heart perfusion system [126]. Treatment with wild-type (WT-) MSC improved left ventricular recovery, while TLR-2 KO MSC did not. This is possibly caused by the lower MSC proliferation rate for the TLR-2 KO, as well as a reduction in vascular endothelial growth factor (VEGF) secretion [126]. A second study performed by the same group had a similar set-up, but used a TLR-4 KO rat heart [118]. Cardioprotection was enhanced in the TLR-4 KO heart, mediated by increased activation of STAT-3. These two studies suggested TLR2 presence and activation could be essential for cardiac recovery, while TLR4 activation would have harmful effects. Unfortunately, as this model contained no immune system, the effect of TLR2 and TLR4 activation on immunomodulation by MSC remains unclear. Others used poly(I:C) preconditioning on MSC before injection into a hamster model of heart failure [133]. The TLR3-preconditioned MSC secreted more IL-6, VEGF, hepatocyte growth factor (HGF), and stromal derived factor 1 (SDF-1) and more proliferating CD34/GATA4 positive progenitor cells were found. Meanwhile, infiltrated immune cells were reduced in number and cardiac function was significantly improved [133]. These outcomes correspond with the *in vitro* studies which suggested TLR3 could boost the MSC immunosuppressive potential, while TLR4 has negative influence of cardiac recovery.

It has been observed that cell injections 7 days after MI give slightly (but not significantly) better outcomes [40]. It is as of yet unclear what might be the cause of this. One of the possibilities is of course the reduced release of DAMPs and therefore a different polarization of the MSC. However, several DAMPs, such as HSP70, remain elevated for at least 14 days [134]. Meanwhile, many other factors could also play a role in the improved results. The healing phase will have started after a week. MSC could influence processes at play at this time after MI, such as scar formation and angiogenesis. It is plausible that DAMPs also influence these processes, although this has not yet been investigated.

## 5. Conclusions

The inflammatory response after MI is essential to initiate reparative pathways and clear debris, yet these activated immune cells cause a lot of short and long term damage to the myocardium. Broad immunosuppressive drugs were only detrimental by reducing both the damaging and healing pathways. Stem cell therapy after MI could improve cardiac function, most likely by the production of paracrine factors. One of the systems influenced by these paracrine factors is the immune system. Basically every immune cell was reported to be affected by MSC in different degrees and subtypes are induced which in turn can influence other immune cell functioning. The mechanisms by which MSC achieve these effects remain unclear, with many groups supporting various effector molecules and pathways. In all studies, however, MSC can influence the immune system via different pathways, thereby having a range of possible effects on their target cells. One of the obvious reasons why different outcomes are still observed is due to the heterogeneity of the MSC and the differences in donor, origin, isolation, culture, and coculture conditions with immune cells. A broad definition for MSC has been defined, but this does not mean all these cells are identical. MSC from different origins can have different capacities and can react differently to similar stimuli [131]. Even when cells are isolated from identical origins according to a strict protocol, strong variations still exist between donors [109, unpublished own observations]. Likewise the timing, concentration, and duration of the stimulation with TLR-ligands can influence observed effects, and the outcome after one hour of stimulation might be entirely different from results after a day of stimulation [124].

Additionally, immunosuppression assays show a lot of variation. Some groups worked with peripheral blood mononuclear cells (PBMC), while others worked with isolated fractions of CD3+ or CD4+ T-cells. It is difficult to compare these results directly with each other, for in a PBMC mixture many other immune cells are present which influence their environment, as shown in Figure 1. Add to this that the immune-suppressing effects on PBMC or T-cell proliferation in the untreated groups varied strongly between groups, it becomes clear that universal protocols are needed to perform this type of assays. In addition, the experiments need to be performed with various different MSC and immune cell donors to make the outcomes more robust.

Although a great effort has been undertaken to identify the effects of TLR activation on MSC, many inconsistencies still remain. Despite the many contradicting reports, some similarities can be found and some clues provided insights into possible mechanisms. Many groups have established the expression of TLR by MSC, although at protein level they are sometimes hard to detect. The effect of TLR activation on proliferation is probably minimal, while differentiation can be interfered with. Although few studies have looked at migration, improved migration might help honing in immunomodulative stem cell therapy and should be investigated further. Initial reports indicate an increase in migration, at least in the acute phase [124]. Regarding the immunomodulatory capacities of MSC, much ambiguity remains. *In vitro* and *in vivo* work seem to indicate that TLR3 activation with poly(I:C) can boost the immunosuppressive potential of MSC, while TLR4 activation with LPS could reduce it. Experimental studies showed TLR2 and TLR4 become activated after MI and are correlated to ischemia-reperfusion injury and LV dysfunction [135–137]. The TLR4 activation can create an unfavorable environment for MSC, reducing their effectiveness as immunomodulatory therapeutics after MI. This in turn would make preconditioning of MSC by using TLR3 ligands to boost immunomodulation an interesting target. These divergent effects of TLR3 and TLR4 signaling have prompted Waterman et al. to suggest MSC can be polarized into inflammatory and anti-inflammatory subtypes by differential TLR activation [124]. However, due to the many contradictory findings, more research will be necessary to validate this hypothesis.

The vast majority of the studies discussed in this review did not focus on cardiac inflammation, but on auto-immune diseases or organ transplantation. This justifies the work with PAMPs, as the main concern will be an infectious threat to a patient with a suppressed immune system. In the setting of inflammation after myocardial infarction, the inflammatory signals consist of DAMPs. It is unlikely that DAMPs and PAMPs activate the same receptors in exactly the same way. There are likely more receptors (PRRs) on the MSC that can recognize these ligands and it could very well be a combined activation of receptors that leads to the activation of a specific pathway in the cell, which could differ between PAMPs and DAMPs. To study the effectiveness of MSC therapy for post-MI inflammation, it would be advisable to investigate the effect of TLR activation on MSC using DAMPs that are released after MI. Only by investigating it this way can the role of TLR activation on MSC in the cardiac setting be truly elucidated.

## Abbreviations

MSC: Mesenchymal stem cell
T_H_: T-helper cell
T_C_: Cytotoxic T-cell
T_reg_: Regulatory T-cells
iDC: Immature dendritic cell
DC: Dendritic cell
Mo: Monocyte
M1: Classically activated type 1 macrophage
M2: Alternatively activated type 2 macrophage
PMN: Polymorphonuclear cells
NK-cell: Natural killer cell
IDO: Indoleamine-pyrrole 2,3-dioxygenase
PGE2: Prostaglandin E2
PD-1: Programmed death 1
PD-L1/2: Programmed death ligand 1 or 2
TGF-*β*: Transforming growth factor *β*.
